# Disseminated tuberculosis and diagnosis delay during the COVID-19 era in a Western European country: a case series analysis

**DOI:** 10.3389/fpubh.2023.1175482

**Published:** 2023-05-18

**Authors:** Sílvia Roure, Xavier Vallès, Nieves Sopena, Rosa Maria Benítez, Esteban A. Reynaga, Carmen Bracke, Cora Loste, Lourdes Mateu, Adrián Antuori, Tania Baena, Germán Portela, Judith Llussà, Clara Flamarich, Laura Soldevila, Montserrat Tenesa, Ricard Pérez, Elsa Plasencia, Jordi Bechini, Maria Lluïsa Pedro-Botet, Bonaventura Clotet, Cristina Vilaplana

**Affiliations:** ^1^Unitat de Salut Internacional Metropolitana Nord, PROSICS Metropolitana Nord, Badalona, Spain; ^2^Direcció Clínica Territorial de Malalties Infeccioses i Salut Internacional de Gerència Territorial Metropolitana Nord, Barcelona, Spain; ^3^Fundació Lluita Contra les Infeccions, Hospital Germans Trias i Pujol, Badalona, Spain; ^4^Germans Trias i Pujol Research Institute, Badalona, Spain; ^5^Servicio de Enfermedades Infecciosas, Hospital Universitari Germans Trias i Pujol, Badalona, Spain; ^6^Microbiology Department, Northern Metropolitan Clinical Laboratory, Hospital Germans Trias i Pujol, Badalona, Spain; ^7^Servei de Radiodiagnòstic de l’Hospital Universitari Germans Trias i Pujol, Badalona, Spain; ^8^Direcció Clínica de Diagnòstic per la imatge de la Gerència Territorial Metropolitana Nord, Badalona, Spain; ^9^Departament de Salut, Subdirecció General de Vigilancia i Resposta a Emergències de Salut Pública, Barcelona, Catalonia, Spain; ^10^Universitat Autònoma de Barcelona, Barcelona, Catalonia, Spain; ^11^Centro de Investigación Biomédica en Red de Enfermedades Respiratorias (CIBERES), Madrid, Spain; ^12^Unitat de Tuberculosi Experimental, Microbiology Department, Germans Trias i Pujol, Badalona, Spain

**Keywords:** disseminated tuberculosis, tuberculosis, delayed diagnosis, hard-to-reach populations, clinical presentation

## Abstract

**Background:**

Disseminated tuberculosis is frequently associated with delayed diagnosis and a poorer prognosis.

**Objectives:**

To describe case series of disseminated TB and diagnosis delay in a low TB burden country during the COVID-19 period.

**Methodology:**

We consecutively included all patients with of disseminated TB reported from 2019 to 2021 in the reference hospital of the Northern Crown of the Metropolitan Area of Barcelona. We collected socio-demographic information, clinical, laboratory and radiological findings.

**Results:**

We included all 30 patients reported during the study period—5, 9, and 16 in 2019, 2020, and 2021 respectively—20 (66.7%) of whom were male and whose mean age was 41 years. Twenty-five (83.3%) were of non-EU origin. The most frequent system involvement was central nervous system (*N* = 8; 26.7%) followed by visceral (*N* = 7; 23.3%), gastro-intestinal (*N* = 6, 20.0%), musculoskeletal (*N* = 5; 16.7%), and pulmonary (*N* = 4; 13.3%). Hypoalbuminemia and anemia were highly prevalent (72 and 77%). The median of diagnostic delay was 6.5 months (IQR 1.8–30), which was higher among women (36.0 vs. 3.5 months; *p* = 0.002). Central nervous system involvement and pulmonary involvement were associated with diagnostic delay among women. We recorded 24 cured patients, two deaths, three patients with post-treatment sequelae, and one lost-to-follow up. We observed a clustering effect of patients in low-income neighborhoods (*p* < 0.001).

**Conclusion:**

There was a substantial delay in the diagnosis of disseminated TB in our study region, which might impacted the prognosis with women affected more negatively. Our results suggest that an increase in the occurrence of disseminated TB set in motion by diagnosis delay may have been a secondary effect of the COVID-19 pandemic.

## 1. Introduction

Disseminated tuberculosis (dTB) is defined as the identification or isolation of *Mycobacterium tuberculosis* or compatible histopathology obtained from two non-contiguous organs in a single patient, from blood or bone marrow specimens and/or a chemotherapeutic response in clinical or image-based compatible cases with multiple foci (1). It is primarily due to the lymph-haematogenous dissemination of *M. tuberculosis* from a pulmonary focus or, less frequently, from another primary TB focus. Disseminated TB accounts for <2% of TB cases and up to 20% of extra pulmonary TB cases (2, 3) but the incidence is modulated by the population-level prevalence of recognized risk factors, especially HIV/AIDS (3–5). It is a severe TB presentation, with much higher mortality compared to classical pulmonary TB [as high as 31.1% (4)] or non-disseminated extrapulmonary TB (6), and similar to TB meningitis (7, 8) which is by large the most severe TB form with unique focus. Like extrapulmonary TB forms, the diagnosis of dTB can be elusive due to the frequently non-specific (like fever of unknown origin) and pleomorphic clinical presentations, lack of clear focus in the early phases of the disease, and its sub-acute or chronic evolution (9, 10). In turn, diagnosis delay is associated to dissemination of TB, as it has been reported in HIV positive patients (11) and be by itself a prognostic factor (12). The diagnosis may be hampered by the difficult access to sampling sites and is frequently based on image findings (13). A limited availability of diagnostic tools and lack of clinical awareness may lead to an underestimation of the disease, which is frequently encountered as an autopsy finding (14). One factor rarely examined is the delayed diagnosis of the primary focus before dissemination occurs. In some instances, such a delay in diagnosis may be due to the quality of the health system (i.e., inadequate coverage, resources, or awareness), which could be aggravated by disruptions of health care services such as occurred during the COVID-19 pandemic and observed among other health conditions (15–17). Socio-cultural barriers to access to care among migrant or low-resourced populations can also be a factor (18, 19). Here we present a detailed analysis of a series of dTB patients that occurred in the metropolitan area of Barcelona, Spain, from 2019 prior to the onset of the COVID-19 pandemic, to the end of 2021. We aim to examine the link between diagnosis delay and the presentation of dTB, to ascertain the underlying risk factors and to undertake a detailed analysis of clinical, image, and laboratory findings of this life-threatening form of TB in a low-endemic country.

## 2. Materials and methods

### 2.1. Study setting and design

This study consisted of a retrospective analysis of all new dTB cases diagnosed at the Hospital Universitari Germans Trias i Pujol in Badalona, Spain, from January 2019 to December 2021. This is the referral hospital for the Northern Crown of the Metropolitan area of Barcelona, the northern urban crown of Barcelona, with 406,000 inhabitants. Immigrants constitute roughly 17% of the population (20). For all new dTB patients reported, we collected socio-demographic, clinical, and laboratory data including patient age, sex, Body Mass Index (BMI), country or region of origin, current household location, and primary and secondary TB foci at diagnosis and laboratory and image findings. Additionally, we noted the date of the first contact with the health system at which symptoms compatible with the initial TB focus diagnosed, and the number of visits between the first recorded symptoms and final TB diagnosis.

### 2.2. Data management and statistical methods

Disseminated tuberculosis was defined as the involvement of TB infection in two or more non-contiguous organs in a single patient. TB infection was determined by either (i) identification or isolation of *M. tuberculosis* or compatible histopathology in samples obtained from lesions of any location or (ii) positive clinical response upon specific treatment in clinical and/or image-based cases compatible with TB infection once other alternative diagnostics had been excluded. Conclusive diagnosis of TB was established by the direct culture of *M. tuberculosis* in conventional media, direct observation in tissue samples (Ziehl-Neelsen or auramine), and means of molecular biology (GenXpert®). Or clinical-based when clinical, laboratory, image findings (RX, RMN, echography, or TC scan) were compatible with TB infection and therapeutic response was positive upon specific treatment. We classified patients with dTB according to the system involved as central nervous system (CNS), gastrointestinal (GI), musculoskeletal (MSK, including bone, muscle, or joints), pulmonary (including pleural), or visceral (involving liver, spleen, peritoneum, or pericardium). Ganglionic involvement was considered separately. We considered the system involvement as initial clinical focus from a clinical perspective, when the given organ or system produced the main guiding symptoms for diagnosis, irrespective of the diagnostic site (i.e., from which the diagnostic sample was obtained). In contrast, we refer to primary focus as the first focus of the dTB disease from a pathophysiology perspective, which is most frequently difficult to ascertain. Data were collected from the electronic records of patients. The clinical electronic records are a backbone of the health system in Catalonia. It is a shared electronic clinical history accessible by all medical and health providers to the Public health system of the region, with a standard interface. Data are fully accessible for research purposes including previous visits before diagnostic, clinical background, symptoms, number of visits and underlying clinical conditions. Data were analyzed using Stata version 14.0 (StataCorp LLC, https://www.stata.com) and R version 4.1.2 (The R Project for Statistical Computing, https://www.r-project.org) software. For descriptive analysis, we used medians and interquartile ranges (IQRs) for continuous variables and proportions and CI: 95% Confidence Intervals (CI) for categorical variables. For univariate analysis, we used the χ^2^ test to compare categorical variables and for trends, when appropriate, or nonparametric Fisher or Wilcoxon tests, when necessary. We performed a geo-location of index cases with cross-reference to estimated mean annual household income in Euros in 2020 obtained from the Spanish government’s National Statistics Institute of Statistics in Spain (21) and then we carried out a multi-distance spatial cluster analysis. We considered a value of *p* ≤ 0.05 significant.

### 2.3. Ethical issues

The present study forms part of the STAGE-TB project, which has been approved by the Ethics Board of the Hospital Universitari Germans Trias i Pujol (study approval code: PI-17-064).

## 3. Results

### 3.1. General description

**Figure 1:**
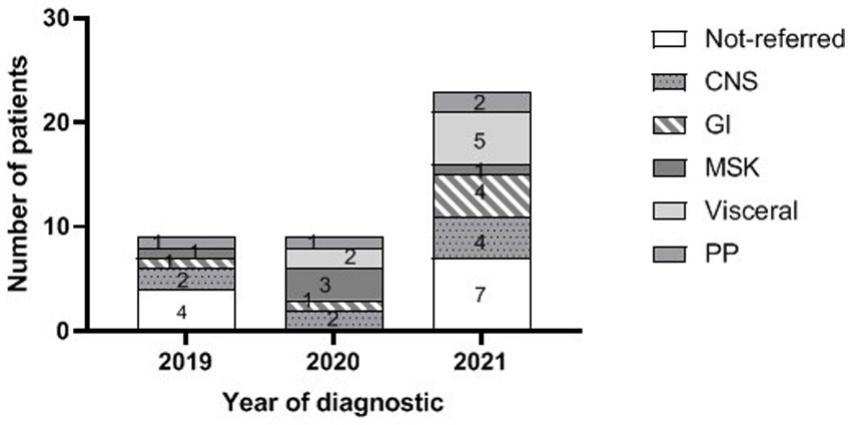
Number of cases by year and initial focus at diagnosis including dTB cases not referred to the HUGTiP (PP, pleuropulmonary; MSK, musculoskeletal; GI, gastrointestinal; and CNS, central nervous system). System involvement refers to the initial TB focus at diagnosis.

Between January 2019 and December 2021, a total of 41 patients with dTB were diagnosed with 9, 9, and 23 cases in 2019, 2020, and 2021, respectively (Table 1; Figure 1), from which 5, 9, and 16, respectively (*N* = 30) where referred to the study Hospital. Twenty (66.7%) were males. The median age was 41 years (IQR 28.8–48.5, range 18–70), without significant differences between sexes (*p* = 0.6). Four of the 10 females were in the puerperium period. The most frequent region of origin was South Asia (India, Pakistan, or Bangladesh, *n* = 17, 56.8%), followed by North-Africa (*n* = 5, 16.7%), and Spain (*n* = 5, 16.7%). The median period of time that migrant patients (*N* = 25) had lived in Spain was 10 years (IQR 5.3–16, range 1–22). The most frequent initial focus at diagnosis was CNS (*N* = 8; 26.7%) followed by Visceral (*N* = 7; 23.3%), GI (*N* = 6, 20.0%), MSK (*N* = 5; 16.7%), and pleuropulmonary (*N* = 4; 13.3%). Conclusive diagnosis was established by laboratory means in 23 out of 30 patients (76.7%), two of them with a *M. tuberculosis* strain with multi-drug resistance. Two patients were associated with a new diagnosis of HIV with a CD4 count <100, four patients (13.3%) had diabetes mellitus type II, three patients (10%) were associated with alcohol consumption, and one case had renal insufficiency. The most frequent organ involvement, including initial and secondary diagnosed foci was pleuropulmonary (*N* = 18; 60.0%), all with abnormal chest X-ray (CXR) findings, including four with a miliary pattern (22.2%). The median diagnostic delay was 6.5 months (IQR 2–30, range 1–108), and the median number of prior visits was 4–5 (IQR 1–8, range 1–15). At the moment of diagnostic 21 out of 29 (72.4%) had low levels of albumin (<3.5 g/dL), including three patients (10.3%) with clinically significant hypoalbuminemia (<2.5 g/dL). Twenty patients (76.9%) met the criteria for anemia (<12.1 g/dL among women and < 13.8 g/dL among men). The median BMI at diagnosis was 24.4 (IQR 20.6–27.6, range 15.8–36.2), and four patients met the criteria for undernutrition (BMI < 18.5).

**Table 1:** General description of the study sample.

Variable	Overall (*N* = 30)	2019 (*N* = 5)	2020 (*N* = 9)	2021 (*N* = 16)	*p*^1^
Median age (IQR)	41 (29–49)	39 (28–45)	30 (25–45)	46 (34–61)	0.06
Males (*N*, %)	20 (66.7)	2 (40.0)	5 (55.6)	13 (81.3)	0.07
Females (*N*, %)	10 (33.3)	3 (60.0)	4 (44.4)	3 (18.8)	
Organ system involvement (*N*, %)					
*Initial clinical focus at diagnosis (N, %)*					
CNS	8 (26.7)	2 (40.0)	2 (22.2)	4 (25.0)	0.6
GI	6 (20.0)	1 (20.0)	1 (11.1)	4 (25.0)	0.4
MSK	5 (16.7)	1 (20.0)	3 (33.3)	1 (6.3)	0.1
Visceral	7 (23.3)	—	2 (22.2)	5 (31.3)	0.3
Pleuropulmonary	4 (13.3)	1 (20.0)	1 (11.1)	2 (12.5)	0.6
*Overall involvement*					
CNS	10 (33.3)	2 (40.0)	4 (44.4)	4 (25.0)	0.4
GI	6 (20.0)	1 (20.0)	1 (11.1)	4 (25.0)	0.7
MSK	7 (23.3)	2 (40.0)	3 (33.3)	2 (12.5)	0.2
Visceral	13 (43.3)	0 (0.0)	2 (22.2)	11 (68.8)	0.004
Pleuropulmonary	18 (60.0)	5 (100)	2 (22.2)	11 (68.8)	0.5
Ganglionic	19 (63.3)	2 (40.0)	6 (66.7)	11 (68.8)	0.7
*Region of origin* (*N*, %)					
South Asia	17 (56.7)	3 (60.0)	4 (44.4)	10 (62.5)	0.5
North Africa	5 (16.7)	1 (20.0)	2 (22.2)	2 (12.5)	0.6
Sub-Saharan Africa	2 (6.7)	1 (20.0)	1 (11.1)	—	0.2
South America	1 (3.3)	—	1 (11.1)	—	0.5
Spain	5 (16.7)	—	1 (11.1)	4 (25.0)	0.3
*Delay in diagnosis* (*median, IQR*)					
By months	6.5 (1.8–30)	24 (12.5–66)	4 (1.5–78)	6 (1.3–11.5)	0.2
By number of visits	5 (1–8)	11 (5–13)	6 (1–9)	3 (1–7)	0.04

1 *p* value comparing 2021 to 2019–2020.

At the time of analysis, 24 (80%) patients had successfully completed treatment with clinical recovery, 2 (7.7%) had died, 2 (6.7%) had neurological sequelae, and 1 (3.9%) had constrictive pericarditis (3.9%). The remaining patient was reported as lost to follow-up. Descriptive data for patients are shown in Table 1. Supplementary Table S1 in Appendix 1 summarizes the main findings case by case, including initial and secondary diagnosed foci and driving symptoms at diagnosis.

### 3.2. Association analysis of dTB patients, TB foci, and studied variables

**Figure 2:**
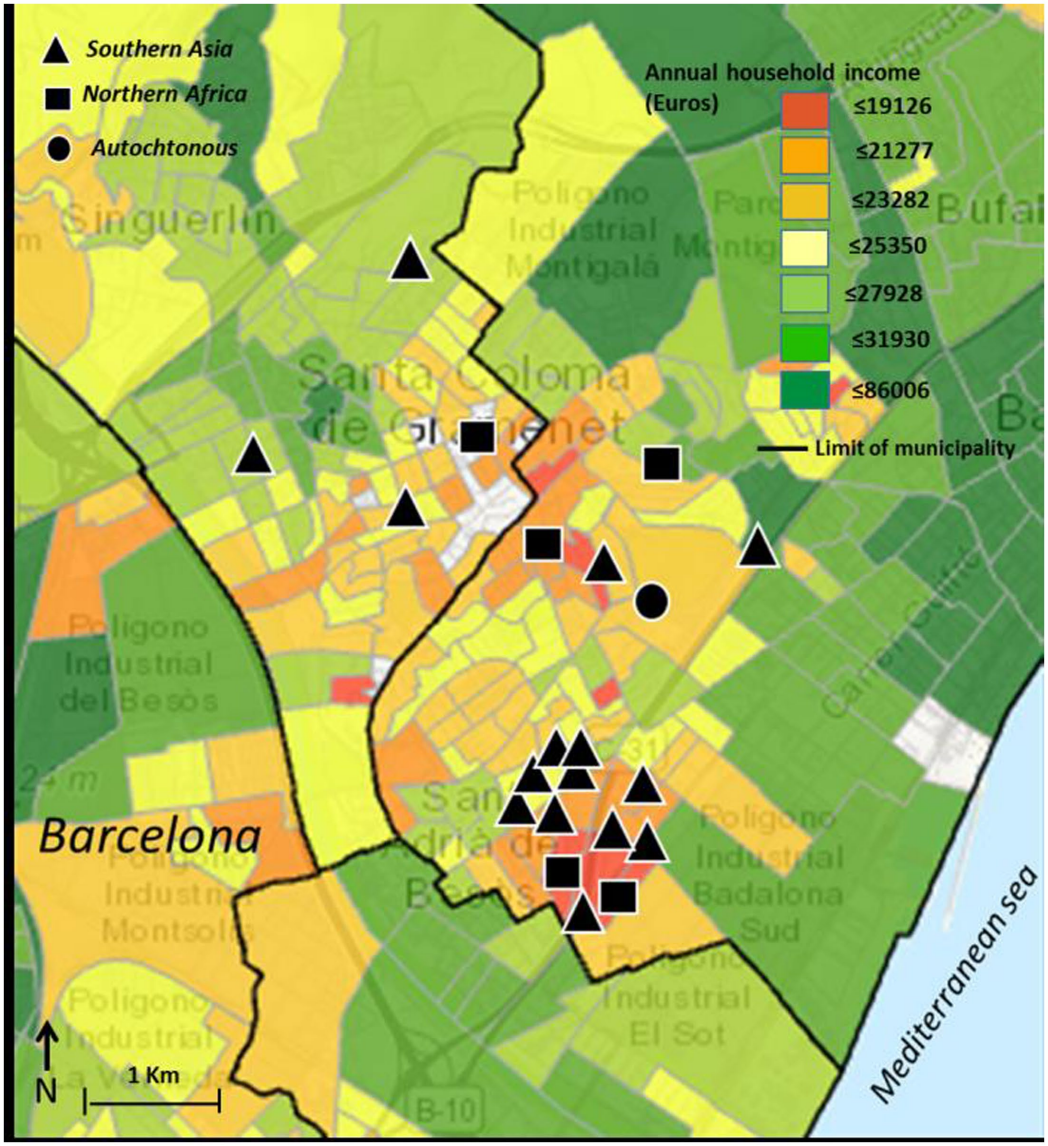
Map showing geo-location of TB disseminated cases in the northern crown of the greater Barcelona metropolitan area (*N* = 23). Patient’s region of origin is indicated by the type of symbol. Symbols have been placed very approximately to protect patient anonymity. Household income map was obtained from (17).

**Figure 3:**
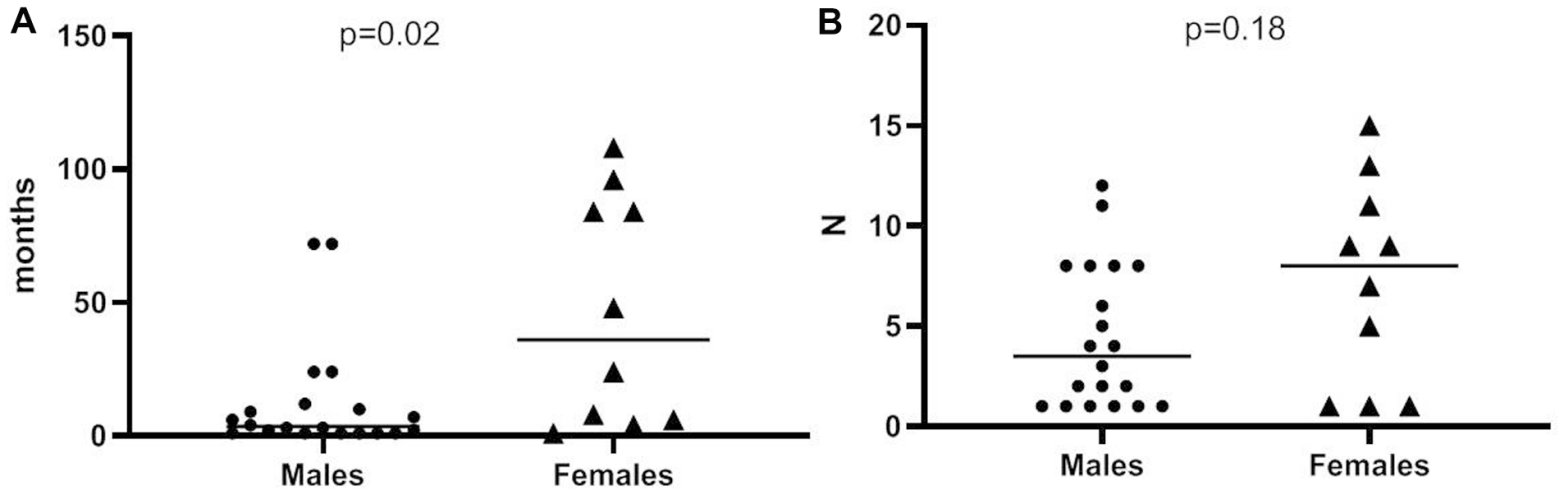
Plot of diagnostic delay (months) stratified by sex **(A)** and by number of visits prior to diagnosis **(B)**. Horizontal bar indicates the median.

**Figure 4:**
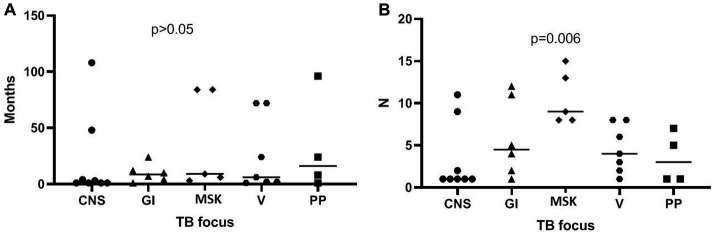
Plot of diagnostic delay (months) stratified by TB initial focus at diagnosis **(A)** and by number of visits prior to diagnosis **(B)** (CNS, central nervous system; GI, gastrointestinal; MSK, musculoskeletal; V, visceral; and PP, pleuropulmonary). Horizontal bar indicates the median.

Patient data for geographic location and socio-economic status showed a clustering pattern (*p* < 0.001), which was especially prominent among patients of South Asian origin (see Figure 2). All patients initially diagnosed as visceral were males (7/20 vs. 0/10; *p* = 0.008) whereas all patients initially diagnosed as pleuropulmonary were females (0/20 vs. 4/10; *p* = 0.04). There was a significant increase in overall visceral involvement in 2021 compared to the 2019/2020 period [11 out of 13 (68.8%) vs. 5 out of 17 (31.3%); *p* = 0.004]. No differences in terms of age at diagnosis, sex, years spent living in the EU, BMI, and other laboratory findings. Diagnosis delay was more prominent among females compared to males [median 36.0 months (IQR 5.5–87) vs. 3.5 months (IQR 1–11.5); *p* = 0.02], but the number of prior visits was not (median 8 [IQR 1–12] vs. 4 [IQR 1–12], *p* = 0.18, see Figure 3). The number of prior visits decreased over time (2019–2020 vs. 2021; *p* = 0.04, see Table 1). There were no significant differences regarding diagnostic delay and initial clinical focus at diagnosis. However, the number of previous visits was significantly higher among patients with an initial MSK focus [MSK focus median 9 visits (IQR 8–14) vs. all other foci 3 visits (IQR 1–8); *p* = 0.006, see Figure 4]. Otherwise, we found an association between diagnosis delay among females with CNS involvement as initial diagnosis focus (*p* = 0.03) or overall CNS involvement (*p* = 0.01). Females with overall pleuropulmonary involvement were associated as well with diagnostic delay (*p* = 0.02).

## 4. Discussion

We observed a sharp increase in the number of dTB over the 2019–2021 period, which is difficult to regard as random oscillation. This trend may reflect the impact of the COVID-19 pandemic, which in many parts of the world has resulted in a substantial reduction in TB testing and access to TB health services (22). The impact of the pandemic on TB surveillance and diagnosis in Catalonia has been well-documented (23). The fact that fewer visits prior to TB diagnosis took place in 2021 compared to 2019–2020 may be precisely due to the more difficult access to diagnostic and care services during the COVID-19 period.

More striking is the clustering of dTB patients around a low-income area, which represents a small section of the study region. It could be argued that this merely reflects the tendency of migrant communities (coming from high TB-burden countries and therefore with higher chances of developing the infection), to share the same neighborhood. However, our data suggest a more complex interaction. Firstly, migrant communities tend to be low-resourced populations with associated risk factors for TB transmission (i.e., overcrowding). According to a previous study, local reinfection is more common among long-term migrants like those from our sample, and is associated with social risk (24, 25). The long-term residence in the EU supports this notion. Secondly, they are more prone to develop poverty-related underlying conditions for TB diseases, such as undernutrition, alcohol abuse, and diabetes mellitus (25). Thirdly, they commonly experience socio-cultural barriers to access to health services (18, 19, 25). All of these factors may increase the risk of TB disease. The development toward a disseminated TB form will be facilitated by delayed diagnosis of the primary focus. Although this last hypothesis should be further explored (13), it is supported in our study sample by, first, the high prevalence of pulmonary involvement, which is *a priori*, an easier form of TB to diagnose and the most frequent primary focus before dissemination. And second, the increasing degree over time of visceral involvement with more atypical TB locations, which might correspond to late stages of TB dissemination expedited by the delayed diagnosis. In this regard, our study underscores the incidence of delayed diagnosis of TB in our study sample, which is much higher than what has been reported for overall TB cases in Catalonia region during the 2019–2022 period (52 days) (23). These delays were preceded by frequent visits to the primary and secondary health care services. The unspecific symptoms reported, language barriers, and the lack of TB awareness on the part of clinicians might have been contributors. Overall, the stressors experienced by our study sample were similar to those reported for a series of HIV-negative migrants with dTB (26). Women may experience the factors described with higher intensity. The delay in diagnosis tends to be more pronounced for women with pulmonary involvement, which is a more easily diagnosable TB form than it is for men, according to a recent study in Portugal (27). Women’s access to healthcare is limited and often dependent on their husbands, and they are likely to face greater language barriers (28). The high prevalence of hypoalbuminemia may be a risk factor for developing TB by itself but likely also a late consequence of delayed diagnosis. Therefore, various factors may overlap with reverse causality effects to favor the development of dTB: social aspects, individual level factors, barriers to healthcare access, and the difficulty of diagnosing extrapulmonary TB. COVID-19 pandemic may have exacerbated these underlying factors through a more difficult access to primary health care (lower number of visits) and the exacerbation of poverty-related conditions. Overall, this could explain the increase of dTB cases over time once other confounding factors have been excluded (i.e., the reference of cases from other facilities overwhelmed by COVID-19), with a differential effect regarding sex. The high prevalence of hypoalbuminemia and anemia among our study sample indicates significant physical deterioration in our patients at the time of diagnosis.

The association of TB with pregnancy puerperium has been described in the literature, showing poor outcomes for both women and their infants, even in low-incidence countries (29–31). It might explain the high proportion of female patients in the post-partum period (four out of 10).

Our case series showed a high incidence of morbidity and mortality, including two deaths, three patients which required intensive care, two patients with serious neurological sequelae and one case with constrictive pericarditis. This morbidity and mortality burden could be avoided with prompt treatment. In the event of TB suspicion and considering the risk–benefit, the initiation of presumptive treatment should be imposed (2).

Finally, the occurrence of dTB in a well-defined pocket of the migrant population should be considered an index event of other health issues, which may deserve further attention like diagnosis of other sub-acute and chronic conditions.

As a main limitation, our small series of dTB patients does not allow us to draw conclusive generalizations and should be confirmed with a pooled analysis of dTB patients from other low TB burden countries during the same period. Secondly, we cannot exclude some bias since not all dTB patients notified in the study area were referred to the reference hospital (11 out of 41). However, the trends and associations observed here might well be representative of other urban areas of Western Europe, and need consideration.

In conclusion, we suggest that TB should be actively ruled out when confronting migrants with recurrent insidious headache, abdominal pain, or back pain, with or without nonspecific chronic symptoms or unexplained weight loss and several prior visits. This is now especially relevant, since it is expected that the major impact of COVID-19 on TB incidence and mortality would be much larger in 2022 and beyond (32). Supplementary Figure S1 in Appendix 2 includes a chart for clinicians describing the prototypical profiles of patients studied which might help them to screen extra-pulmonary of dTB. We provide as well radiological images in the Supplementary material (Appendix 2). Otherwise, the occurrence and delayed diagnosis of disseminated TB could be tempered with interventions aimed at pockets of vulnerable populations, such as raising awareness among clinicians or lowering barriers to access to health services with a gender perspective. Such strategies could mitigate the contributing background conditions such as malnutrition and overcrowding of underserved migrant communities and strengthen the resilience of the health system in the face of socio-economic crises.

## Data availability statement

The data analyzed in this study is subject to the following licenses/restrictions: data were obtained from clinical records of patients and are subject to confidentiality. It could not be shared to personnel outside the research institution (Hospital Universitari Germans Trias i Pujol). Requests to access these datasets should be directed to ceic.germanstrias@gencat.cat.

## Ethics statement

The studies involving human participants were reviewed and approved by Ethics Board of the Hospital Universitari Germans Trias I Pujol, study approval code: PI-17-064. Written informed consent for participation was not required for this study in accordance with the national legislation and the institutional requirements.

## Author contributions

SR, XV, NS, RB, ER, CB, CL, LM, AA, TB, GP, JL, CF, LS, MT, RP, EP, JB, MP-B, BC, and CV: study design, data curation, statistical analysis, supervision, and article writing. All authors contributed to the article and approved the submitted version.

## Funding

This work was supported by the Catalan Government (2021 SGR 00920), the Spanish Government-FEDER Funds (CPII18/00031 and PI20/01424), the CIBER Enfermedades Respiratorias (CB06/06/0031), and European Union’s Horizon 2020 research and innovation program (847762).

## Conflict of interest

The authors declare that the research was conducted in the absence of any commercial or financial relationships that could be construed as a potential conflict of interest.

## Publisher’s note

All claims expressed in this article are solely those of the authors and do not necessarily represent those of their affiliated organizations, or those of the publisher, the editors and the reviewers. Any product that may be evaluated in this article, or claim that may be made by its manufacturer, is not guaranteed or endorsed by the publisher.
