# Corrigendum: Disseminated tuberculosis and diagnosis delay during the COVID-19 era in a Western European country: a case series analysis

**DOI:** 10.3389/fpubh.2023.1236874

**Published:** 2023-06-28

**Authors:** Sílvia Roure, Xavier Vallès, Nieves Sopena, Rosa Maria Benítez, Esteban A. Reynaga, Carmen Bracke, Cora Loste, Lourdes Mateu, Adrián Antuori, Tania Baena, Germán Portela, Judith Llussà, Clara Flamarich, Laura Soldevila, Montserrat Tenesa, Ricard Pérez, Elsa Plasencia, Jordi Bechini, Maria Lluïsa Pedro-Botet, Bonaventura Clotet, Cristina Vilaplana

**Affiliations:** ^1^Unitat de Salut Internacional Metropolitana Nord, PROSICS Metropolitana Nord, Badalona, Spain; ^2^Direcció Clínica Territorial de Malalties Infeccioses i Salut Internacional de Gerència Territorial Metropolitana Nord, Barcelona, Spain; ^3^Fundació Lluita Contra les Infeccions, Hospital Germans Trias i Pujol, Badalona, Spain; ^4^Germans Trias i Pujol Research Institute, Badalona, Spain; ^5^Servicio de Enfermedades Infecciosas, Hospital Universitari Germans Trias i Pujol, Badalona, Spain; ^6^Microbiology Department, Northern Metropolitan Clinical Laboratory, Hospital Germans Trias i Pujol, Badalona, Spain; ^7^Equip Atenció Primària Sant Roc, Institut Català de la Salut, Badalona, Spain; ^8^Servei de Radiodiagnòstic de l'Hospital Universitari Germans Trias i Pujol, Badalona, Spain; ^9^Direcció Clínica de Diagnòstic per la imatge de la Gerència Territorial Metropolitana Nord, Badalona, Spain; ^10^Departament de Salut, Subdirecció General de Vigilancia i Resposta a Emergències de Salut Pública, Barcelona, Catalonia, Spain; ^11^Universitat Autònoma de Barcelona, Barcelona, Catalonia, Spain; ^12^Centro de Investigación Biomédica en Red de Enfermedades Respiratorias (CIBERES), Madrid, Spain; ^13^Unitat de Tuberculosi Experimental, Microbiology Department, Germans Trias i Pujol, Badalona, Spain

**Keywords:** disseminated tuberculosis, tuberculosis, delayed diagnosis, hard-to-reach populations, clinical presentation

In the published article, there was an error in affiliation of “Adrián Antuori”. Instead of “Equip Atenció Primària Sant Roc, Institut Català de la Salut, Badalona, Spain”, it should be “Microbiology Department, Northern Metropolitan Clinical Laboratory, Hospital Germans Trias i Pujol, Badalona, Spain”.

In the published article, an author name was incorrectly written as Adrià Antuori. The correct spelling is Adrián Antuori.

The authors apologize for this error and state that this does not change the scientific conclusions of the article in any way. The original article has been updated.

