# Supplementary material for: Disseminated tuberculosis and diagnosis delay during the COVID-19 era in a Western European country: a case series analysis
Source: Front Public Health. 2023 May 18;11:1175482. doi: 10.3389/fpubh.2023.1175482 (PMC10233202; doi:10.3389/fpubh.2023.1175482)
Supplement: Supplementary file 1 [file Data_Sheet_1.docx]

This supplementary material is hosted by the Journal of Travel Medicine as supporting information alongside the article “Disseminated tuberculosis and delayed diagnosis during the Covid-19 era in a Western European country: a case series analysis”, on behalf of the authors, who remain responsible for the accuracy and appropriateness of the content. The same standards for ethics, copyright, attributions and permissions as for the article apply.

**Figure S1:** Prototypical profiles of patients who should be checked for dTB

**
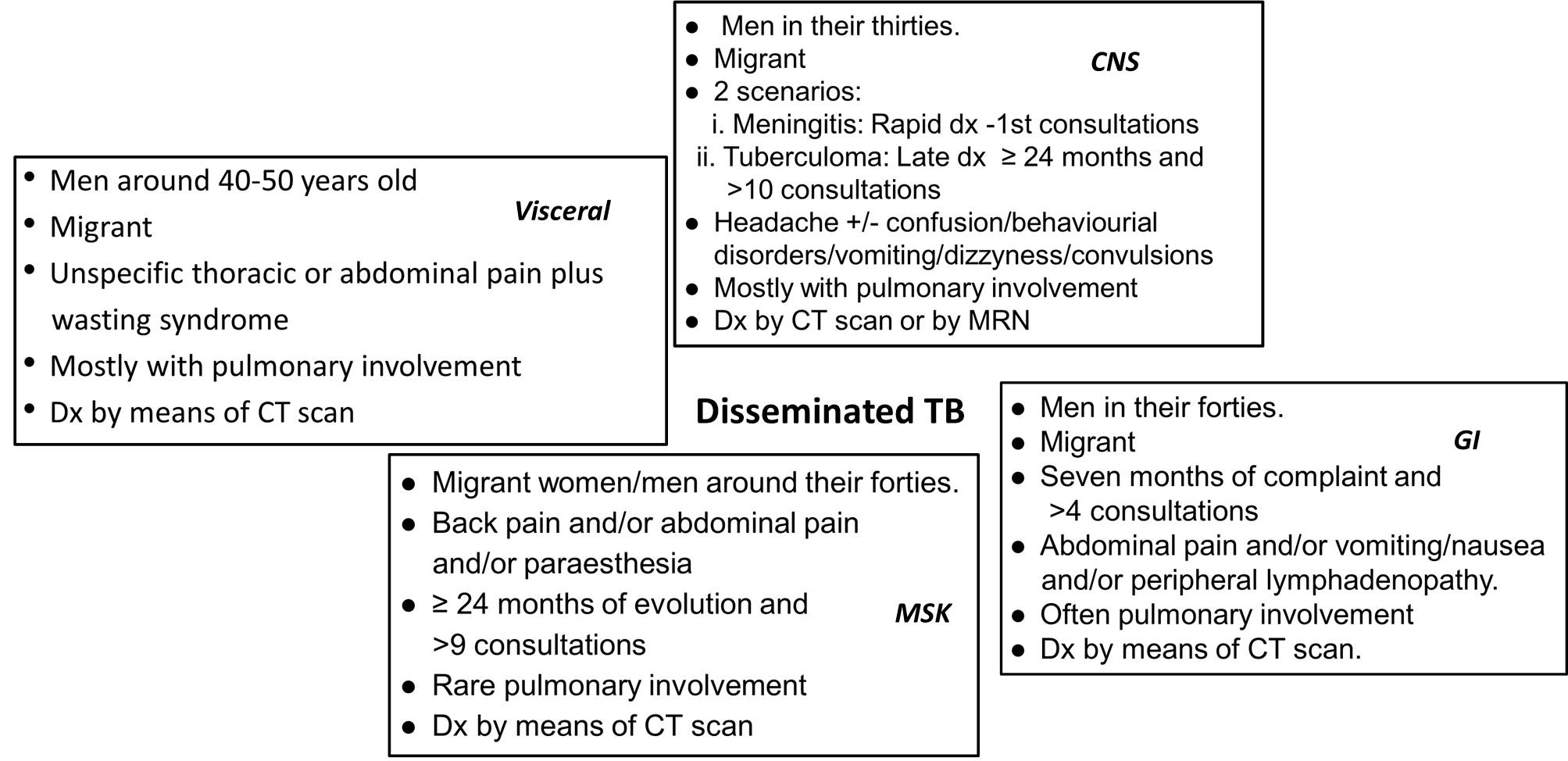
**

**Diagnostic images characterising prototypical patient profiles according to the primary focus of dTB**

**CNS-Focus dTB**


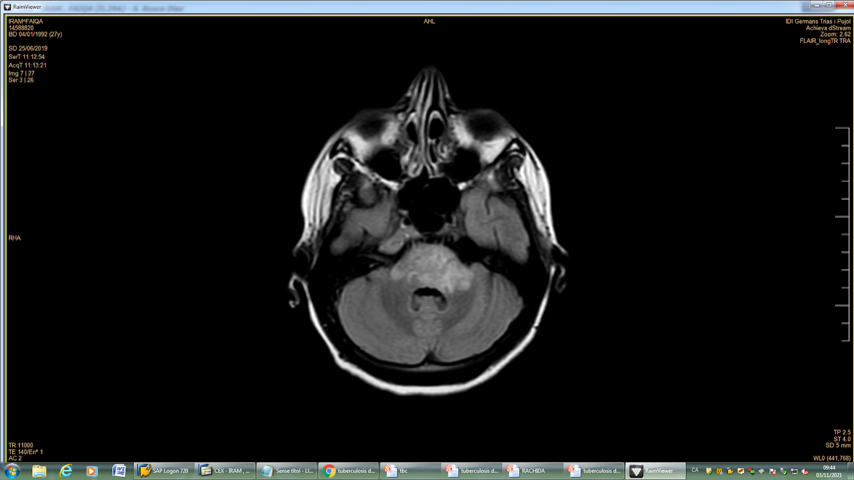


Figure A. Cranial CT: Leptomeningitis, subacute ischemic haemorrhagic infarcts, frontal, parietal, occipital parenchymal lesions, cerebellar lesions.


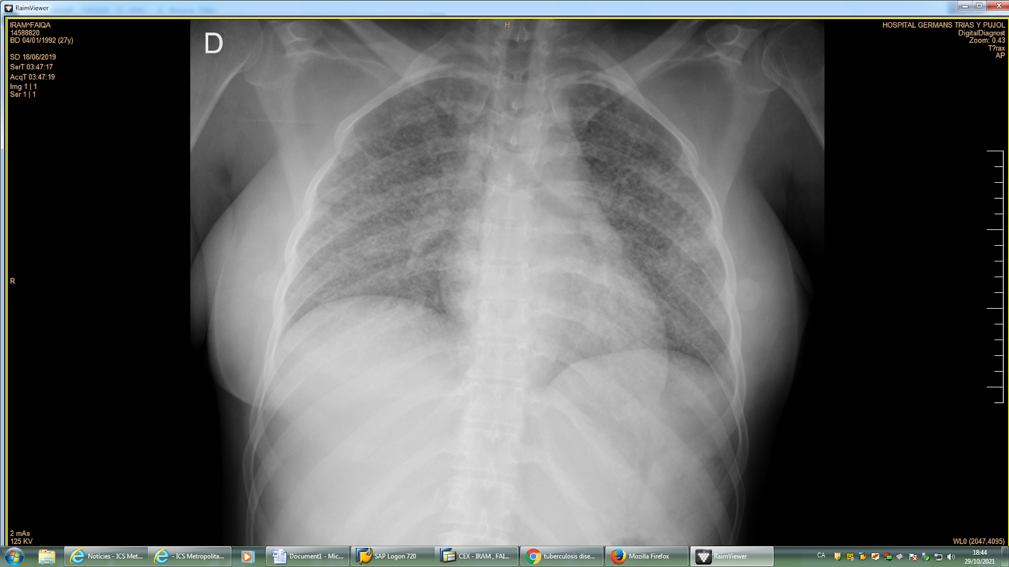


Figure B. CT: Miliary + hilar, mediastinal adenopathies.


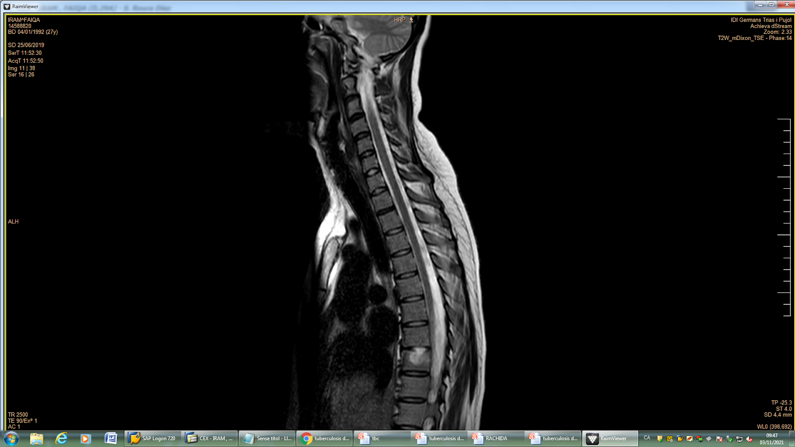


Figure C. T7-T8 spondylodiscitis.

**MSK-focus dTB**


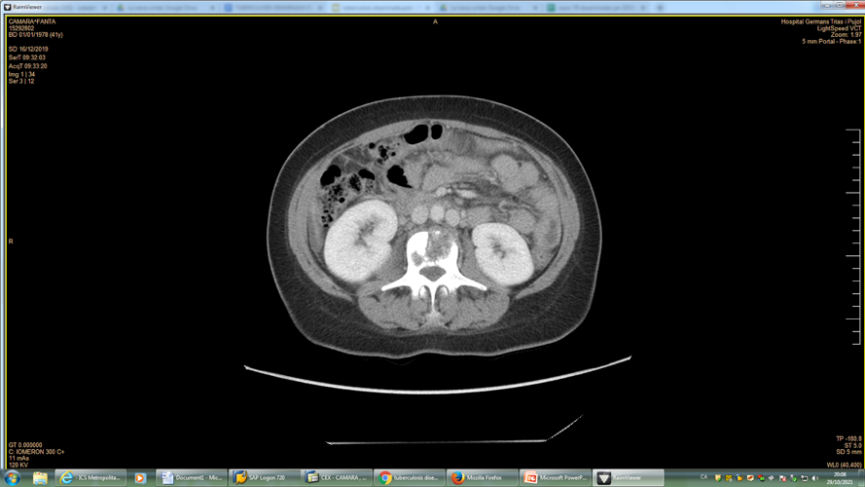


Figure D. Lumbosacral MRI: multiple bone lesions in T12, L2, L3, L4, L5, sacrum and right iliac bone (1) and paravertebral abscess (2)


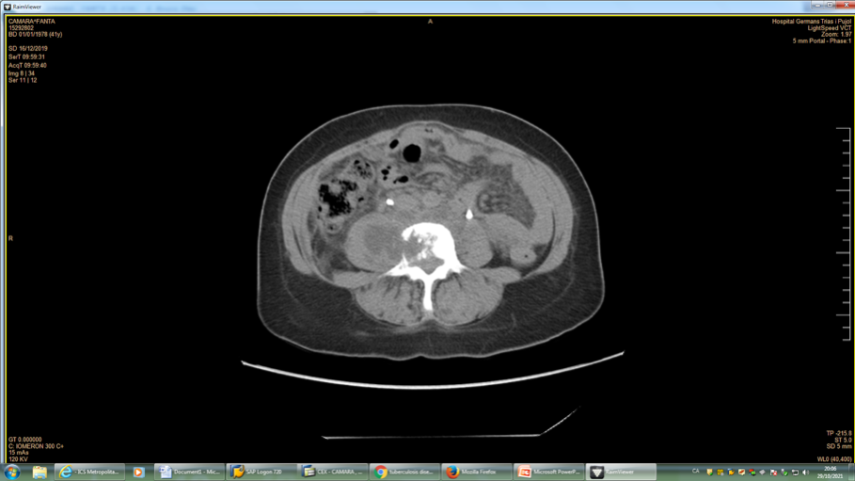


Figure E. Insufflation of L4-L5 causing spinal (1) canal stenosis and paravertebral abscesses (2)


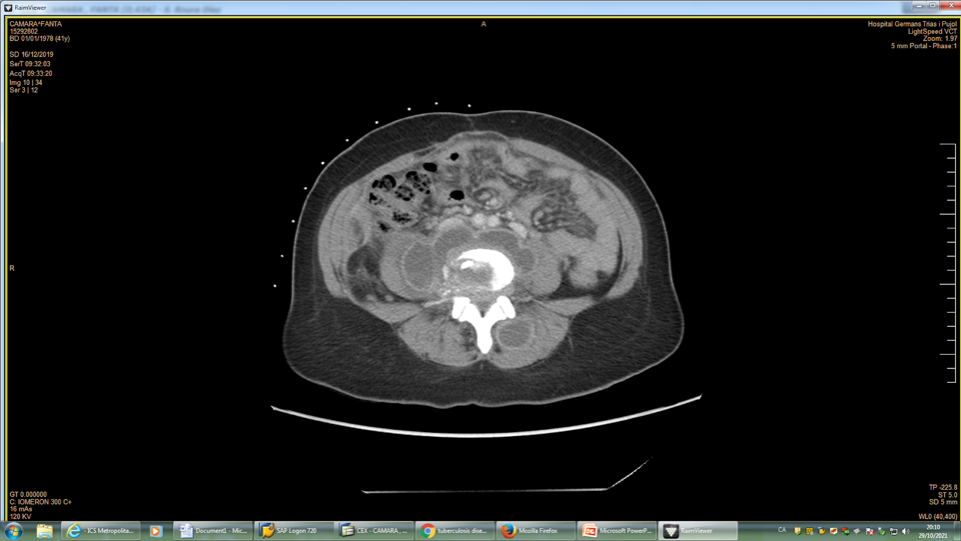


Figure F. Bone destruction S1 vertebra (1), soft tissue involvement (2)


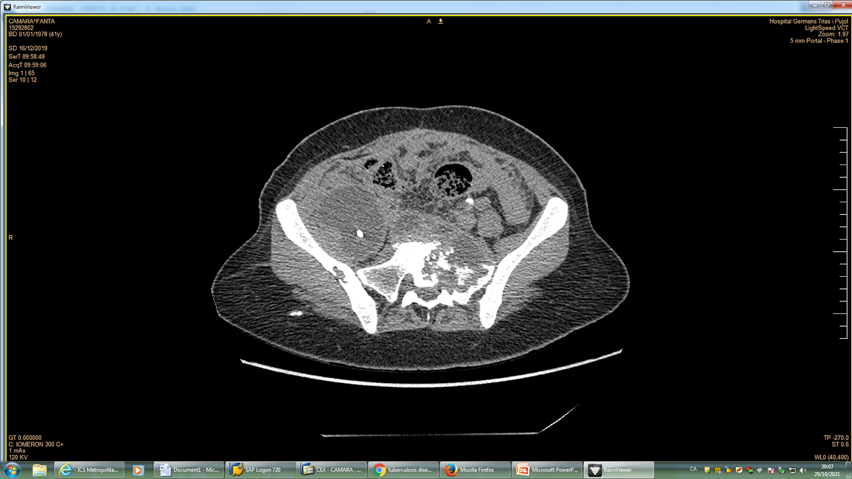


Figure G. Psoas muscle abscess dr, prevertebral collection.

:


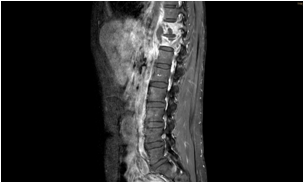


Figure H. Discitis D11-D10 (1), fluid collection towards the intervertebral space, collection of 47 × 23 mm in left prevertebral space (2).


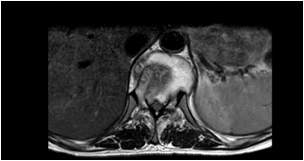


Figure I. Lytic lesion in iliac bone dr exceeds the posterior cortex towards gluteal musculature. Cystic lesions in pelvis suggestive of ovarian adnexal lesions.


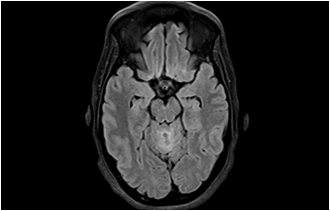


Figure J. MRI cr: cerebellar vermix abscesses.

**GI-focus dTB**


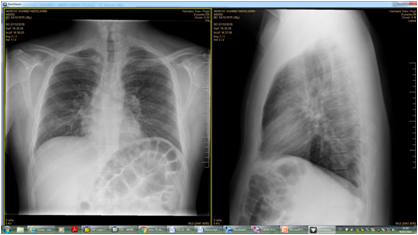


Figure K. Chest X-ray: Parahilar infiltrate dr, LID and possible LII.


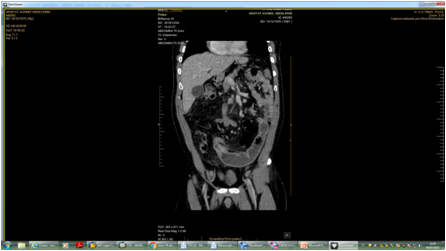


Figure I: CT-Scan: Iliar obstruction

**Visceral-focus dTB**

**
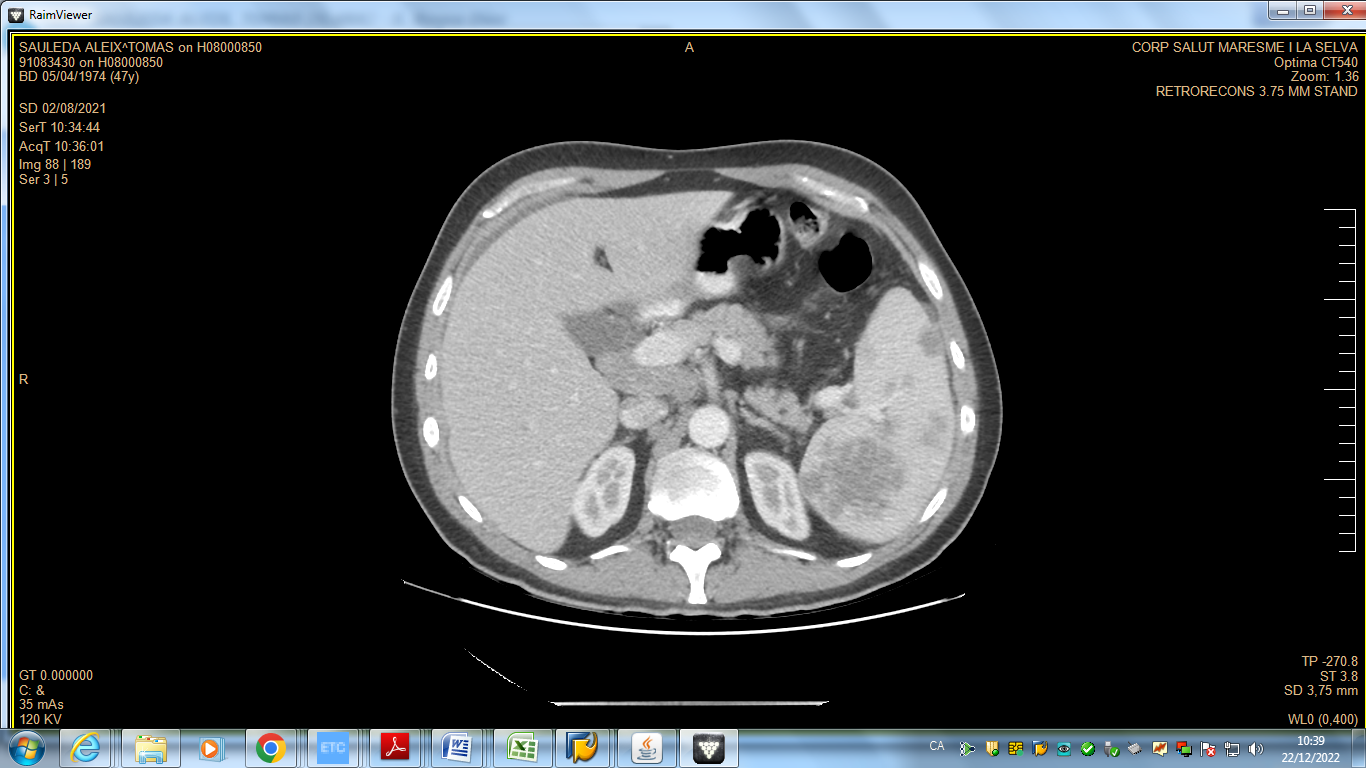
**

Figure N. **Contrast-enhanced CT** Spleen, retroperitoneal and vertebral involvement

**
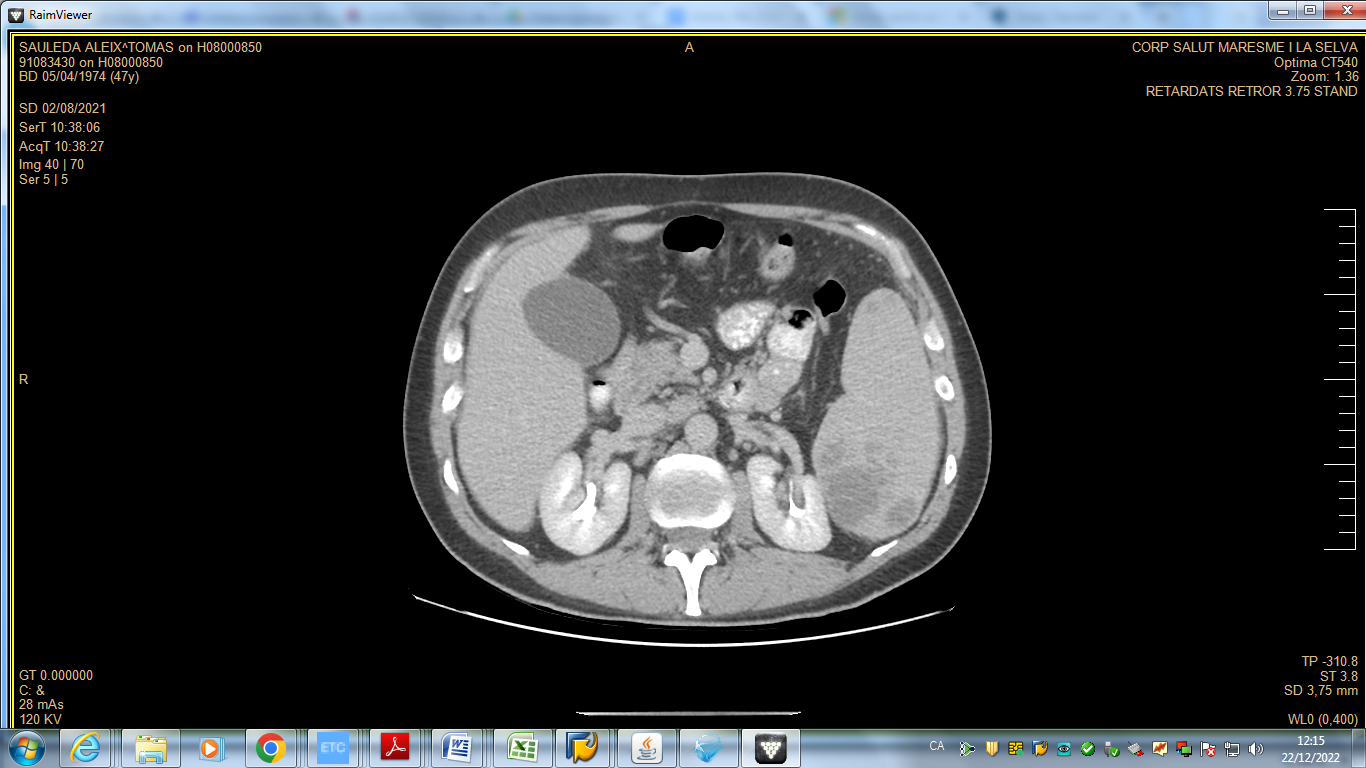
**

Figure O. **Contrast-enhanced CT:** Splenomegaly with multiple round non enhancing low-density splenic lesions (CC axis of approximately 16cm) without calcification, with the presence of multiple confluent hypodense LOES, with infiltrative aspect.

**
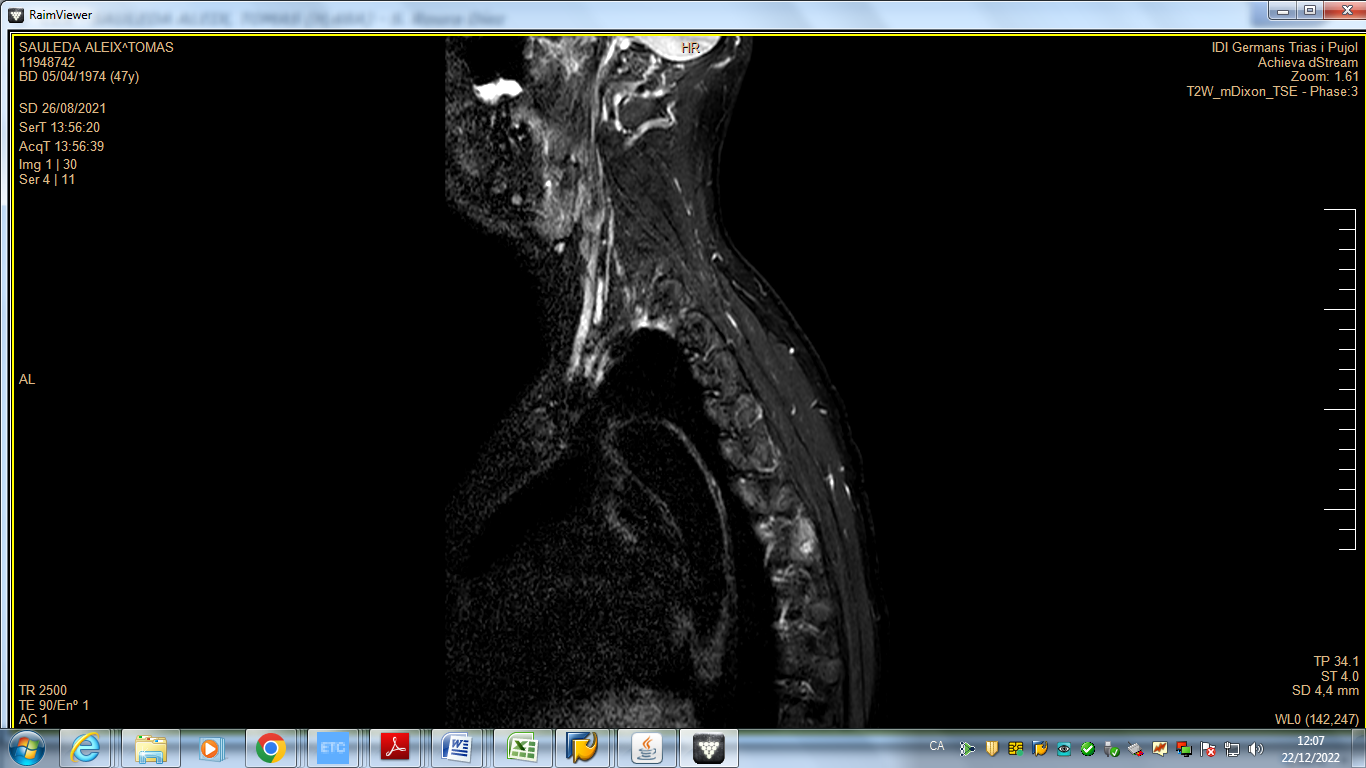
**

Figure P. **Dorsolumbar spine MRI:** Focal lesion in the posterolateral aspect of the medial portion of the left transverse apophysis at T7, hyperintense on T2-weighted sequence and hypointense on T1-weighted sequence correlating with the small osteolytic focus
