# Supplementary material for: Disseminated tuberculosis and diagnosis delay during the COVID-19 era in a Western European country: a case series analysis
Source: Front Public Health. 2023 May 18;11:1175482. doi: 10.3389/fpubh.2023.1175482 (PMC10233202; doi:10.3389/fpubh.2023.1175482)
Supplement: Supplementary file 2 [file Table_1.DOCX]

This supplementary material is hosted by Journal of Travel Medicine as supporting information alongside the article “Disseminated tuberculosis and delayed diagnosis during the Covid-19 era in a Western European country: a case series analysis”, on behalf of the authors, who remain responsible for the accuracy and appropriateness of the content. The same standards for ethics, copyright, attributions and permissions as for the article apply.

**Table S1:** Detailed clinical description of the patients

| **Case no.** | **Primary focus** | **clinical form** | **Age (yrs.) and sex** | **Driving symptom a diagnosis** | **Secondary focus except lung and ganglionic** | **Visceral adenopathy** | **Pulmonary involvement** | **Diagnosis** |
| --- | --- | --- | --- | --- | --- | --- | --- | --- |
| **1** | CNS | Cerebral and medullary tuberculum | 27, male | Paraparesis | Retroperitoneal and hepatic | No | Yes | Lab confirmed |
| **2** |  | Meningo-encephalitis | 30, male | Fever and confusional state | none | No | No | Lab confirmed |
| **3** |  | Meningo-encephalitis | 66, male | Aphasia hypoesthesia | Spleen | Yes | Yes (miliary) | Lab confirmed |
| **4** |  | Hypophysitis | 45, female | Headache and diplopia | none | Yes | No | Clinical |
| **5** |  | Meningo-encephalitis | 33, male | Wasting syndrome, fever and photophobia | Ileitis and peritonitis | No | Yes | Lab confirmed |
| **6** |  | Tuberculoma and Meningo-encephalitis | 27, female | Headache, bradipsychia and vomiting | Vertebral and paravertebral abscess | No | Yes (miliary) | Lab confirmed |
| **7** |  | Meningo-encephalitis | 38, male | Vomiting and respiratory distress | none | Yes | Yes (miliar) | Lab confirmed |
| **8** |  | Cerebral tuberculoma with hydrocephalus | 29, male | Headache, vomiting and unconsciousness | none | No | Yes | Lab confirmed |
| **9** | GI | Ileitis | 24, male | Abdominal pain | none | No | Yes | Lab confirmed |
| **10** |  | Ileitis | 51, male | Abdominal pain and diarrhoea | Sacroiliac bone | Yes | No | Clinical |
| **11** |  | Ileitis and colitis | 27, female | Supraclavicular adenopathy | none | Yes | Yes | Lab confirmed |
| **12** |  | Ileitis and colitis | 70, male | Fever and cough | Peritoneum | Yes | Yes | Lab confirmed |
| **13** |  | Ileitis | 40, male | Abdominal pain and acute hepatitis | Peritoneum and hepatic | No | Yes | Lab confirmed |
| **14** |  | Ileitis | 39, male | Abdominal pain, adenopathy and lower respiratory tract infection symptoms | none | Yes | Yes | Lab confirmed |
| **15** | MSK | Dorsal spondylitis | 45, female | Headache, dizziness and back pain | Cerebral abscess, gluteal abscess and ovaries | No | No | Lab confirmed |
| **16** |  | Lumbar/sacral spondylitis | 47, female | Back pain | Iliopsoas muscle, retroperitoneal and paravertebral lumbar abscesses | No | No | Lab confirmed |
| **17** |  | Dorsal spondylitis | 42, female | Cough and back pain | Tube-ovarian and Iliopsoas muscle abscess | No | Yes | Lab confirmed |
| **18** |  | Cervical spondylitis | 30, male | Neck pain with irradiation to right hand and ataxia | Intraspinal abscess | No | No | Lab confirmed |
| **19** |  | Dorsal spondylitis | 18, male | Back pain, myalgia and paraesthesia | Mediastinum | Yes | No | Lab confirmed |
| **20** | Visceral | Peritonitis | 37, male | Abdominal pain | Colon, liver and spleen | Yes | Yes (miliary) | Lab confirmed |
| **21** |  | Prostatitis | 45, male | Fever of unknown origin and prostatitis | Liver | Yes | Yes | Lab confirmed |
| **22** |  | Pericarditis | 61, male | Fever, dyspnoea | none | Yes | Yes | Clinical |
| **23** |  | Retroperitoneal | 44, male | Abdominal pain | Ureter | Yes | No | Clinical |
| **24** |  | Peritoneal | 63, male | Supraclavicular adenopathy  New HIV diagnostic | None | Yes | Yes | Lab confirmed |
| **25** |  | Pericarditis | 22, male | Cough, fever and loss weight | Mediastinum, liver | No | Yes | Lab confirmed |
| **26** |  | Peritoneal | 47, male | Abdominal pain and loss weight | Spleen, retroperitoneal and vertebral | Yes | No | Lab confirmed |
| **27** | Pleuropulonary | Pleural | 60, female | Cough | Panniculitis | Yes | Yes | Clinical |
| **28** |  | Pleural | 28, female | Chest pain | Pericardium | Yes | No | Clinical |
| **29** |  | Lung | 48, female | *Erythema nodosum* | Panniculitis | Yes | Yes | Clinical |
| **30** |  | Lung | 50, female | Chest pain and dyspnoea;  New HIV diagnosis | Oesophagus | Yes | Yes | Lab confirmed |
